# Analyzing emergency call volume, call durations, and unanswered calls during the first two waves of the COVID-19 pandemic compared to 2019: An observational study of routine data from seven bavarian dispatch centres

**DOI:** 10.1016/j.heliyon.2024.e24839

**Published:** 2024-01-23

**Authors:** Florian Dax, Moritz Waibel, Katharina Kneißl, Stephan Prückner, Marc Lazarovici, Florian Hoffmann, Kathrin Hegenberg

**Affiliations:** a Institut für Notfallmedizin und Medizinmanagement (INM), Klinikum der Universität München, LMU München, Schillerstr. 53, 80336, München, Germany; b Dr. von Haunersches Kinderspital, Kinderklinik und Kinderpoliklinik, Klinikum der Universität München, LMU München, Lindwurmstr. 4, 80337, München, Germany

**Keywords:** Integrated Dispatch Centre, Emergency call, Call volume, COVID-19 pandemic

## Abstract

**Background:**

The spread of the COVID-19 pandemic and the corresponding implementation of measures such as stay-at-home orders and curfews had a major impact on health systems, including emergency medical services. This study examined the effect of the pandemic on call volumes, duration of calls and unanswered calls to the emergency number 112.

**Method:**

For this retrospective, descriptive study, 986,650 calls to seven emergency dispatch centres in Bavaria between January 01, 2019 and May 31, 2021 were analysed. The absolute number of calls and calls per 100,000 inhabitants as well as the number of unanswered calls are reported. The Mann‒Whitney *U* test was used to compare mean call durations between 2019 and 2020/2021 during several periods.

**Results:**

Call volume declined during the pandemic, especially during periods with strict lockdown restrictions. The largest decline (−12.9 %) occurred during the first lockdown. The largest reduction in the number of emergency calls overall (−25.3 %) occurred on weekends during the second lockdown. Emergency call duration increased, with the largest increase (+13 s) occurring during the “light” lockdown. The number of unanswered calls remained at a similar level as before the pandemic.

**Conclusion:**

This study showed that the studied Bavarian dispatch centres experienced lower call volumes and longer call durations during the first two waves of the COVID-19 pandemic (up to May 2021). Longer call durations could be the result of additional questions to identify potentially infectious patients. The fact that the number of unanswered calls hardly changed may indicate that the dispatch centres were not overwhelmed during the study period.

## 1 Introduction

The number of pre-hospital emergency medical services (EMS) responses in Germany has steadily increased for years [1]. The trend in the federal state of Bavaria is similar [2,3]. However, pre-hospital EMS responses represent only a fraction of the chain of events initiated by a medical emergency. At the beginning of the chain, dispatch centres handle requests for help, alert fire services or ambulances, and coordinate the transport of patients to suitable treatment facilities [4]. Hence, the rising number of pre-hospital EMS responses correlates with a rising number of emergency calls. An effective dispatch centre is therefore a prerequisite for adequate handling of medical emergencies. However, the workload of dispatch centres and ambulances are not necessarily directly related, as dispatch centres also perform a filtering function. Callers who seek only information must be referred to appropriate helplines to allow the dispatch centres personnel to be available for medical emergency calls. The dispatch centre plays an important role for the whole pre-hospital EMS system, as callers can be directed to various health care settings [5].

The coronavirus disease 19 (COVID-19) pandemic has affected the utilization of pre-hospital EMS in several ways. In response to the COVID-19 pandemic, the German federal government and the Bavarian state government imposed a set of restrictions to contain the spread, shutting down large parts of public life during several periods. These measures, as well as the fear of infection in the early days of the pandemic, presumably affected the type and frequency of pre-hospital EMS use. Consequently, this change would have altered the utilization of the emergency number (112) and thus the integrated dispatch centres that answered these calls. Moreover, people may have desired more medical information during the pandemic. In response to the pandemic, dispatch centres altered several processes [[6], [7], [8]]. The Bavarian Ministry of the Interior for Sport and Integration issued a directive that the dispatch protocol includes additional questions to identify potentially infected patients [9]. These additional queries likely increased the duration required to process an emergency.

Many analyses have focused on ambulance services; however, the role of the coordinating dispatch centres has received less attention. Some studies have suggested that ambulance dispatch centres faced an increase in calls during the COVID-19 pandemic [7,8,[10], [11], [12], [13], [14], [15]]; in contrast, a decline in the number of emergency calls has also been documented [8,[16], [17], [18], [19], [20]]. This decline in emergency calls is in line with a decrease in the utilization of EMS [21,22] and decreasing numbers of patients admitted to emergency departments [23] at the beginning of the pandemic. A combination of these phenomena has also been reported [24]. However, few studies have focused on the change in the number of emergency calls and the time needed to process these calls.

To the best of our knowledge, no studies to date have examined changes in the number of medical emergency calls and the duration of these calls in Germany during the COVID-19 pandemic. For this study, we extracted and analysed telephone data of calls to the medical emergency number (112) from seven integrated dispatch centres. We investigate changes in the number of calls, the number of unanswered calls and call durations and compared these values to the same period in previous years. The goal of the analyses was to examine the workload of integrated dispatch centres to obtain a better picture of the changes that affected the rescue chain during the pandemic.

## 2 Methods

For this retrospective, descriptive study, data were extracted from the dispatch centres’ telephone systems, which automatically keep track of incoming calls, between January 01, 2019 and May 31, 2021. The seven investigated regional dispatch centres were operated by the Bavarian Red Cross (Bayerisches Rotes Kreuz, BRK). A data usage agreement was concluded with the BRK for the use of the data, which permits the analysis and publication of the data from the dispatch centres operated by the BRK. These integrated dispatch centres can be reached via the European emergency number (112). They coordinate emergency and nonemergency ground and air ambulance responses as well as the fire brigade and alert the appropriate vehicles [25]. In Bavaria, 26 regional dispatch centres cover different areas and coordinate calls and rescue vehicles. Each area covered consists of one or more counties and independent cities. Response decisions are made by the dispatchers, who use a non-standardized, keyword-based dispatch protocol. A guideline is provided to support the decision-making process [26].

Germany's first confirmed COVID-19 case was reported on January 27, 2020, near Munich, Bavaria. This cluster was fully contained, but case numbers subsequently began to increase in March. By May 31, 2021, Germany had undergone several waves of the pandemic. In response to rising case numbers, on March 18, 2020, the Bavarian Ministry of the Interior for Sports and Integration (StMI) issued several guidelines [9]. To identify patients potentially infected with severe acute respiratory syndrome coronavirus 2 (SARS-CoV-2), additional questions were added to the dispatch protocol for patients with nonspecific general symptoms, fever or respiratory symptoms. These questions were as follows.

• Has the patient tested positive for COVID-19?
• Has the patient been in contact with someone with confirmed COVID-19 in the past 14 days?
• Has the patient recently (within the last 14 days) travelled to an international risk area listed by the Robert Koch Institute (RKI)?

All eight dispatch centres operated by the Bavarian Red Cross provided data for this study. The information included when calls were made, when calls were answered, when calls ended, and whether the caller hung up before the call was answered. Therefore, the number of calls, call durations (answer until end of call), and the number of unanswered calls were analysed. Data from one dispatch centre (Donau-Iller) were excluded because the exported data were incomplete. Thus, data from seven dispatch centres were included in the analysis. One dispatch centre (Mittelfranken-Süd) was not able to provide data before September 2019; data from this call centre is therefore not included in analyses comparing the observed (pandemic) periods to corresponding periods in 2019.

The database included 986,650 calls between January 01, 2019 and May 31, 2021. After removing 15 duplicate calls for which values in all columns matched and removing records that could not be assigned a date, the dataset consisted of 986,632 calls. Deviations of the number of calls below the expected call volume were likely due to technical problems that led to incomplete transmissions of call numbers on certain days. We assumed a data gap if a dispatch centre did not receive a call within at least 6 h. We detected data gaps at five dispatch centres over a total of 68 days. On days with data gaps, the number of calls was corrected to the median number of calls on the same day of the week in other weeks of that month if the daily call volume fell below the median call volume of that day of the week and month. Thus, a total of 2778 calls were added to the dataset, and the analyses were based on 989,410 calls. Upwards deviations in call numbers were permissible as events such as thunderstorms, fires or heat waves can lead to high demand on individual days.

Usually, calls are terminated before or upon arrival of the ambulance at the scene. In Bavaria, emergencies should be reached by a paramedic-staffed ambulance no later than 12 min after dispatch. Thus, call durations of more than 15 min were considered implausible. A total of 681 calls (0.07 % of the available calls) lasted longer than 15 min and were truncated at 15 min. Calls of shorter duration were deemed plausible and included in the analyses.

Annual data on the population of Bavaria were provided by the Bavarian State Office for Statistics. For 2021, the population count from 2020 was assumed. The analyses were supplemented with publicly available data on the number of COVID-19 cases [27].

Periods defined by key pandemic-mitigation measures were determined and investigated. To identify these periods, we screened official regulation documents (Bayerische Infektionsschutzmaβnahmenverordnung (BayIfSM) and extracted the relevant dates. The lockdown periods were mainly characterized by rigorous contact restrictions, closed restaurants and shops as well as distance learning or restricted access to schools and daycare centres whereas restrictions were less severe and depended on the incidence of the respective district during the incidence-dependent restriction period. The period between March 20, 2020 and May 10, 2020 was labelled the first lockdown. Incidence-dependent restrictions were in place between May 11, 2020 and November 01, 2020, followed by a “light” lockdown from November 02, 2020 to December 9, 2020. From December 10, 2020, the measures were tightened again, and the second lockdown was in place until April 23, 2021.

Unanswered calls are calls where the caller hung up before the call was answered by a dispatcher. These calls were classified as “unanswered” in the original dataset. The emergency line (112) is subject to stringent safety protocols and compliance with the ‘Technical Guidelines for Emergency Calls’ (Technische Richtlinie Notrufverbindungen -TR Notruf). If all available lines are engaged, a series of technical mechanisms are employed to either temporarily queue the caller or seamlessly redirect them to a nearby dispatch centre. Consequently, instances in which a call is left unattended are virtually eliminated. Nevertheless, it is conceivable that a caller may elect to terminate the call prematurely due to a preference not to endure any waiting period.

The number of calls is expressed as an absolute number or as the number of calls per 100,000 population (the call rate). Depending on the analysis, the rate refers either to calls per day or to calls per a defined time period. These time periods were compared with the corresponding periods in 2019.

The daily number of calls, daily number of unanswered calls, daily median call duration and daily median 7-day incidence of COVID-19 were visualized as line graphs. The absolute number per time period and relative change are presented for the number of calls. Median relative changes ± interquartile range (IQR) are presented for call durations. The Mann‒Whitney *U* test was used to compare the mean call duration of 2019 with that of 2020 and 2021 respectively.

## 3 Results

The fewest calls per day over the entire period occurred on March 05, 2020 (736 calls). The maximum number of calls per day (2455 calls) was observed before the first lockdown on February 10, 2020. The number of unanswered calls per day ranged between 17 (on March 11, 2019) and 284 (on October 25, 2020). A total of 15 % of calls was classified as unanswered. The median duration of answered calls was 0,03 (IQR 0,05) minutes.

**Fig. 1:**
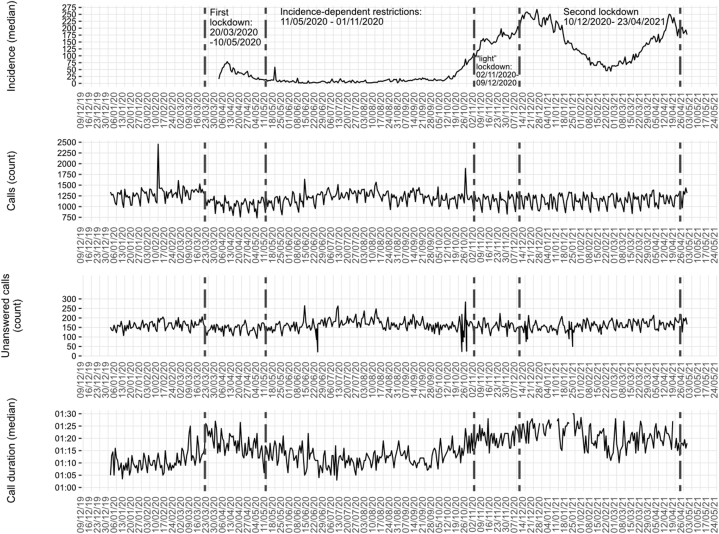
Median number of cases reported in the previous seven days per 100.000 population (incidence), number of calls, unanswered calls and median call duration to the medical emergency number 112.

Fig. 1 shows the daily median number of cases reported in the previous seven days per 100.000 population (incidence) in the areas covered by the dispatch centres in this study as well as the daily number of calls, the daily number of unanswered calls, and daily median call durations. Relevant periods during the SARS-CoV-2 pandemic in 2020 are marked by dashed lines. At the beginning of the first lockdown, there was a decline in the number of emergency calls and a large increase in median call duration. Both of these effects gradually decreased over time and returned to initial levels around the middle of the following period (of incidence-dependent restrictions). Subsequently, the number of calls dropped slightly and remained below the baseline level. Call durations begin to increase again in August (during the period of incidence-dependent restrictions). After a subsequent slight decrease, the median call duration remained markedly above the initial level in the beginning of 2020.

In all examined pandemic periods, a decrease in the number of calls was observed compared to the same period in 2019 (Table 1). The difference was greatest during the first lockdown, with a total decrease of 12.9 %. This decline was most pronounced on Wednesdays and Thursdays; in contrast, an increase in the number of calls of 5.2 % occurred on Saturdays.

**Table 1:**
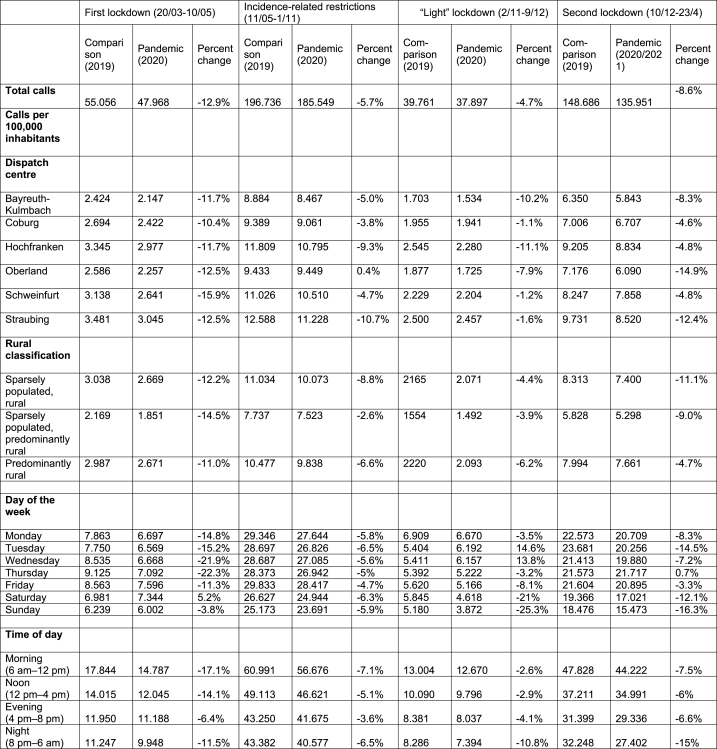
Total number of calls during the observation (pandemic) periods vs. comparison periods (the same period in 2019).

During the period of incidence-related restrictions, the decrease in the number of calls was distributed evenly across all days of the week. In contrast, during the “light” lockdown and the second lockdown, the greatest reductions were observed on weekends, with up to 25.3 % fewer calls recorded on Sundays during the “light” lockdown. Additionally, during the “light” lockdown, up to 14.6 % more calls were recorded on Tuesdays and Wednesdays than in the same period of the previous year.

While the largest decrease in the number of emergency calls during the first lockdown and during the period of incidence-dependent restrictions was observed during the day (6 a.m.–4 pm), the largest decreases in this number during the “light” lockdown and the second lockdown were observed at night (8 p.m.–6 am). The largest decline in the number of calls (−17.1 %) during the first lockdown occurred during the morning (6 a.m.–12 p.m.). In contrast, the largest decline in the number of calls (−15 %) during the second lockdown occurred at night (8 p.m.–6 am).

During the first lockdown, the number of calls exhibited similar decreases among all dispatch centres analysed. Subsequently, greater heterogeneity in the declines in the number of calls (i.e., large vs. small declines) was observed. Moreover, one dispatch centre (Oberland) even reported no change in the number of calls (+0.4 %) during the period with incidence-related restrictions compared to the same period in the previous year.

Call durations are presented in Table 2. Compared to the previous years, a higher median call duration was observed during all periods examined. The smallest difference (+6 s, (p < 0.00)) occurred during the period of incidence-dependent restrictions, and the largest difference (+13 s p < 0.00)) occurred during the “light” lockdown.

**Table 2:**
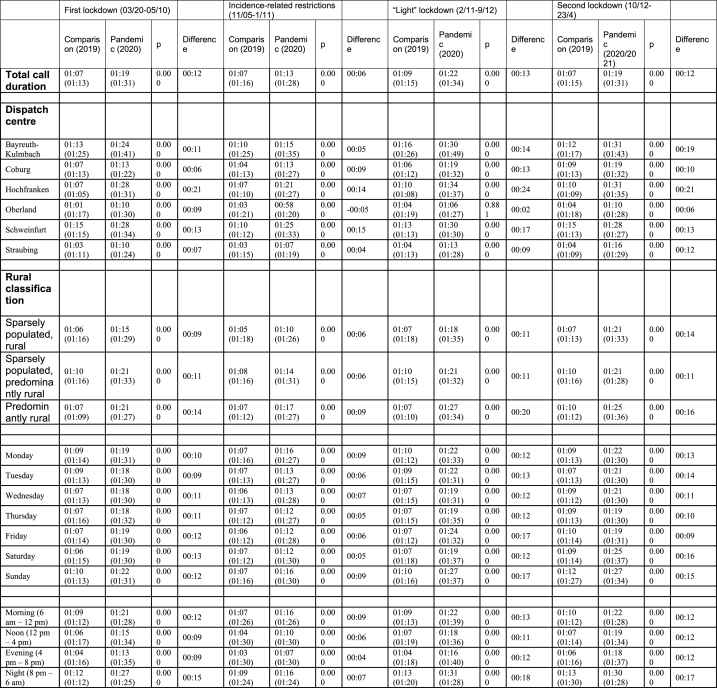
Call duration [median (IQR); in minutes: seconds] during the observation (pandemic) periods vs. comparison periods (the same period in 2019).

The call duration at the Hochfranken dispatch centre was consistently significantly longer than that at all other dispatch centres, except during the period with incidence-dependent restrictions.

In the Oberland dispatch centre, a shorter call duration (−5 s, p = 0.000) was found during the period of incidence-dependent restrictions compared to the two previous years. During the “light" lockdown, there was no significant difference compared to the previous year at this dispatch centre. In the counties with higher population densities, there was a larger increase in call duration in all time periods.

Calls tended to last longer at night than at other times of the day. Similar to differences in the number of calls, the period of incidence-related restrictions was an exception, as the longest calls were registered during the morning in this period.

The number of unanswered calls changed only slightly (Fig. 1). There was an initial slight decline in the number of unanswered calls during the first lockdown. During the middle of the subsequent period with incidence-dependent restrictions, this number returned to the prepandemic level. Subsequently, the number of unanswered calls declined slightly and remained just below the 2019 level for the rest of the year. Descriptively, no correlation with the COVID-19 incidence was apparent.

## 4 Discussion

This study examined call volumes during the first two waves of the COVID-19 pandemic using data from seven integrated dispatch centres in Bavaria. During periods with strict restrictions and curfews (“lockdown"), call volumes to the emergency number (112) decreased. During these same periods, the durations of emergency calls increased; however, the number of unanswered calls remained at a similar level.

### 4.1 Call volume

In Bavaria, several periods had strict restrictions to prevent COVID-19 from spreading. At the beginning of the pandemic (the first lockdown), public life was almost completely shut down throughout Bavaria, and curfews were imposed. The second lockdown was largely similar. Changes in call volume observed during these periods differed from changes observed during the period with incidence-based restrictions. This pattern seems plausible since incidence-based restrictions were only imposed in regional hotspots and not throughout Bavaria.

The greatest decline in call volumes occurred in periods with the most severe restrictions (first and second lockdown). The extent to which contact and mobility restrictions contributed to this decline is unclear. A decreased number of calls is consistent with reduced mobility and reduced availability of recreational activities such as sports and nightlife, which could explain the sharp declines in call numbers during periods with strict restrictions. Several studies have reported declines in the number of emergency calls for traffic accidents and trauma associated with migitation measures [[16], [17], [18],^24^,^28^]. Emergencies involving alcohol were also recorded less frequently [16,17,24,28]. Similar results were reported by Ferron et al. yet the authors also reported an increase in the number of calls regarding substance overdose [18].

Patients avoiding emergency medical services and hospitals because of preceived greater risk of exposure to the virus [29] and challenges in accessing medical advice during lockdowns and movement restrictions might also add to the declines during periods with high COVID-19 incidence.

During the “light” lockdown and second lockdown, the number of calls exhibited the steepest decline on weekends. This finding might be explained by some parts of daily life returning to normal, such as workers returning to offices, but not others, such as recreational opportunities. However, this theory is contradicted by the fact that approximately one-third of the patients receiving pre-hospital EMS care were older than 75 years [3,30], and thus presumably less affected by reductions in recreational opportunities and nightlife scenes.

Several surveys have reported an increase in anxiety levels, especially at the beginning of the pandemic and among people describing their health as “poor" [[31], [32], [33]]. In the prior severe acute respiratory syndrome (SARS) epidemic of 2003, fear of infection led to avoidance of medical services [34]. Delayed access and avoidance of emergency care due to fear of infection has also been reported during the COVID-19 pandemic [29,35]. Thus, fear of infection while seeking pre-hospital EMS or hospital care could also have led to a reduction in the number of calls.

Other countries have reported a sharp increase in call volume [36]. This observation, which is contradictory to our findings, may be partly explained by the fact that our observation period was long, whereas other studies focused on the peaks of the pandemic. In addition, the structure of EMS systems differs among countries. Calls of people seeking medical advice are often handled by dispatch centres as well. Jensen et al. reported that in Copenhagen, a year-to-year comparison between 2020 and 2019 revealed that emergency calls (to 112) increased by 4.4 %; in contrast, calls to the medical advice number increased by 25.1 % [7]. Our analysis included only emergency calls (to 112). However, it is possible that in Bavaria, especially at the beginning of the pandemic, requests for advice were also directed to dispatch centres via the emergency number (112). The actual decline in emergency calls could thus have been even greater than the observed decline. However, increased advertisement for the medical helpline operated by on-call physician services (116,117) [37], likely reduced advice-related calls to the emergency number over the course of the pandemic. Increasing awareness of the number of on-call services, a reduction in unnecessary emergency calls, containment measures (such as entry restrictions at events), and the remaining possibility of working remotely are all possible explanations why the number of calls did not return to prepandemic levels, even by the end of the study period.

The decline in the number of calls probably correlated with the decline in the number of ambulance deployments and was roughly proportional [3]. This finding indicates that there was not increased filtering to separate advice-related calls from emergency calls at the dispatch centres.

### 4.2 Call duration

At the beginning of the pandemic as well as at the end of 2020, an increase in emergency call duration of several seconds was observed. Increased demand for advice is a plausible cause for this increase at the beginning of the pandemic; later in the pandemic, additional questions were added to the emergency call protocol. In a study from Berlin, Dahmen et al. reported that the additional questions took an additional 1:36 min on average [38]. In Bavaria, additional questions were also implemented. These questions presumably led to increases in call durations overall, especially during phases with high COVID-19 incidence. When COVID-19 incidence was low, the additional questions concerning infection and contact with infected individuals are assumed to have been usually answered in the negative. Follow-up questions were thus often less necessary than in times of high incidence. Furthermore, the time spent processing calls at the dispatch centre has increased in Bavaria for many years [39]. The increase in call duration could therefore have occurred independently of the COVID-19 pandemic. This theory is supported by the fact that even during the pandemic, many emergencies that were processed by dispatch centres were not related to COVID-19 and did not come with an increased need for health-related advice.

The Coburg and Hochfranken dispatch centres were comparable in terms of their structure and number of employees. However, the emergency call duration differed at these centres after the outbreak of the pandemic. This difference could indicate different implementation of the additional call questions by the two dispatch centres.

### 4.3 Unanswered calls

The number of unanswered calls (calls in which the caller hung up before being answered by a dispatcher) hardly changed over the course of the pandemic and was also comparable to before the pandemic. Unanswered calls were investigated as an indicator that the capacity of dispatch centres were exceeded. While other studies have reported an increase in daily workload for staff of integrated dispatch centres during the COVID-19 pandemic [36,40], our results suggest that dispatch centres were not overloaded. A possible reason for this lack of overload could be decreased demand. Alternatively, the measures implemented to prepare dispatch centres for the impending challenge could have been effective. Another explanation is that sufficient staff were available because vacations and training sessions were cancelled due to contact restrictions and curfews. Additionally, staff that usually operated nonemergency patient transport could have been freed up because elective and ambulatory procedures were postponed and access to medical rehabilitation services was temporarily restricted. Moreover, employees could have compensated for the increase in calls by working extra hours or by answering emergency calls more quickly, which could explain why employees still reported an increased workload during these periods.

## 5 Limitations

The present study analysed only calls to the emergency number (112). Other service numbers handled by integrated dispatch centres, such as the number for ambulance services (19,222) or direct lines to the police, were not considered. Furthermore, no numbers operated by other parties, such as the numbers for the on-call services (116,117) or health-office advice, were included. It is possible that these service numbers compensated for some of the decrease in emergency calls.

Since calls are registered automatically, the completeness of call records is considered high. Nevertheless, we identified periods of data gaps explained by technical issues. To address these gaps, the number of calls during these periods was estimated. A few calls (0.07 %) that lasted longer than 15 min were truncated, as longer durations than 15 min were implausible. An analysis of the cut-off call durations showed no systematic differences with regard to the dispatch centres as well as the temporal distribution.

The generalizability of these data to other states in the Federal Republic of Germany or to other countries may be low due to differences in EMS systems.

## 6 Summary

This study shows that during the first three waves of the COVID-19 pandemic, dispatch centres experienced lower call volumes but slightly longer call durations than corresponding prepandemic periods. The number of unanswered calls remained largely the same. The slightly longer call durations could be predominantly due to the addition of dispatch protocol questions after changes in the mandatory statutory requirements. Other studies have shown that dispatch centre employees report a higher burden. Yet the lack of change in the number of unanswered calls might indicate that dispatch centres were not stretched beyond their capacity. Indeed, an increase in demand was initially expected, and corresponding countermeasures were initiated; however, contrary to expectations, the use of the emergency number actually declined. The reasons for emergency calls and caller characteristics were not investigated. Further research is needed to elucidate the reasons for the observed changes.

## Data availability statement

The data used in this paper was a combination of data about 112 calls, population data, spatial data, and reported COVID-19 incidence.112 call data are third-party data analysed with permission of the Bayerisches Rotes Kreuz where the authors do not have the permission to share the data. Population data are publicly available from the Bavarian State Office for Statistics. Spatial data are publicly available from the German Federal Institute for Research on Building, Urban Affairs and Spatial Development. Data of confirmed COVID-19 cases are available from the Robert Koch Institute.

## CRediT authorship contribution statement

**Florian Dax:** Writing – review & editing, Writing – original draft, Validation, Methodology, Conceptualization. **Moritz Waibel:** Writing – original draft, Validation. **Katharina Kneißl:** Formal analysis, Data curation. **Stephan Prückner:** Validation, Supervision, Conceptualization. **Marc Lazarovici:** Writing – review & editing, Validation. **Florian Hoffmann:** Writing – review & editing, Supervision. **Kathrin Hegenberg:** Writing – review & editing, Writing – original draft, Validation, Project administration, Methodology, Data curation, Conceptualization.

## Declaration of competing interest

The authors declare the following financial interests/personal relationships which may be considered as potential competing interests:Florian Dax reports a relationship with Bavarian Red Cross that includes: employment. If there are other authors, they declare that they have no known competing financial interests or personal relationships that could have appeared to influence the work reported in this paper.
